# P-278. Assessing the Intersection of Drug Use, Sexual Risk, and HIV/STI Burden: A Surveillance Study in the Dominican Republic

**DOI:** 10.1093/ofid/ofaf695.499

**Published:** 2026-01-11

**Authors:** Rosa Sanchez, Melvin Brioso, Robert Paulino-Ramirez, Maritza Molina, Juan José Polanco

**Affiliations:** National Council for HIV/AIDS CONAVIHSIDA, Santo Domingo, Distrito Nacional, Dominican Republic; National Council for HIV/AIDS CONAVIHSIDA, Santo Domingo, Distrito Nacional, Dominican Republic; Universidad Iberoamericana, Santo Domingo, Distrito Nacional, Dominican Republic; Center for Demographic and Social Studies CESDEM, Santo Domingo, Distrito Nacional, Dominican Republic; Center for Demographic and Social Studies CESDEM, Santo Domingo, Distrito Nacional, Dominican Republic

## Abstract

**Background:**

Drug use and HIV pose a public health challenge in the Dominican Republic (DR), where stigma and limited healthcare access increase risks for PWUD. This study assesses HIV seroprevalence, STIs, and healthcare engagement to inform targeted interventions.

**Methods:**

A cross-sectional study was conducted in five Dominican provinces using respondent-driven sampling to recruit PWUD aged ≥18 who reported drug use in the past six months. Structured interviews collected demographic, behavioral, and healthcare data, while biological samples were tested for HIV, syphilis, HBV, and HCV. HIV-positive individuals were assessed for awareness, ART history, and care engagement.

**Results:**

A total of 2,155 PWUD were recruited across study sites. Most participants were male, with a median age of 36 years (IQR: 30–41). Among respondents, 78% (95% CI: 76.3%–79.7%) reported inconsistent condom use in the past six months. Regarding self-reported sexual orientation, 42% identified as bisexual. The seroprevalence of HIV was 4.0%, while syphilis, HCV, and HBV rates were 11.5%, 1.2%, and 2.7%, respectively. Among PWH, 67% (95% CI: 65.0%–69.0%) engaged in transactional sex for money, goods, or services. Only 16.7% (95% CI: 15.1%–18.3%) had ever initiated ART, and 14.3% (95% CI: 12.8%–15.8%) reported current ART use. Notably, 74% (95% CI: 72.1%–75.9%) of PWUD with prior ART experience had discontinued treatment. Engagement with biomedical HIV prevention strategies was minimal. Only 6.7% (95% CI: 5.6%–7.8%) of PWUD had heard of PrEP, while 3.8% (95% CI: 3.0%–4.6%) had ever used it. PrEP persistence was even lower, with just 2.6% (95% CI: 1.9%–3.3%) reporting use in the past six months.

**Conclusion:**

Findings highlight critical gaps in HIV prevention and care, emphasizing the need for integrated, evidence-based interventions. Expanding PrEP access through harm reduction services, strengthening ART adherence through peer-led models, and integrating harm reduction into national HIV strategies are essential. Addressing stigma and criminalization via policy reforms and scaling up HIV/STI testing and linkage-to-care models is crucial. These findings provide evidence to inform policy and programmatic interventions, supporting global HIV epidemic control.

**Disclosures:**

All Authors: No reported disclosures

